# Nanostructured pencil graphite electrodes for application as high power biocathodes in miniaturized biofuel cells and bio-batteries

**DOI:** 10.1038/s41598-020-73635-7

**Published:** 2020-10-06

**Authors:** Álvaro Torrinha, Nomnotho Jiyane, Myalowenkosi Sabela, Krishna Bisetty, Maria C. B. S. M. Montenegro, Alberto N. Araújo

**Affiliations:** 1 LAQV-REQUIMTE, Laboratório Química Aplicada, Faculdade de Farmácia, Universidade do Porto, Porto, Portugal; 2 Department of Chemistry, Durban University of Technology, P.O Box 1334, Durban, 4000 South Africa

**Keywords:** Chemistry, Electrochemistry, Materials chemistry

## Abstract

This work describes a simple method for the fabrication of an enzymatic electrode with high sensitivity to oxygen and good performance when applied as biocathode. Pencil graphite electrodes (PGE) were chosen as disposable transducers given their availability and good electrochemical response. After electrochemical characterization regarding hardness and surface pre-treatment suited modification with carbon-based nanostructures, namely with reduced graphene, MWCNT and carbon black for optimal performance was proceeded. The bioelectrode was finally assembled through immobilization of bilirubin oxidase (BOx) lashed on the modified surface of MWCNT via π–π stacking and amide bond functionalization. The high sensitivity towards dissolved oxygen of 648 ± 51 µA mM^−1^ cm^−2^, and a LOD of 1.7 µM, was achieved for the PGE with surface previously modified with reduced graphene (rGO), almost the double registered for direct anchorage on the bare PGE surface. Polarization curves resulted in an open circuit potential (OCP) of 1.68 V (vs Zn electrode) and generated a maximum current density of about 650 μA cm^−2^ in O_2_ saturated solution.

## Introduction

Pencil graphite electrodes (PGEs) are valuable analytical tools. As disposable probes, their usefulness ranges from daily routine analysis, up to be embedded in microfluidic systems where are meant for electrochemical detection in proof-of-concept experiments^1^. Although first reported in 1960^2^, PGEs have been used in an increasing number of applications, and particularly as sensors and biosensors since the new millennium^3–6^. In some studies their use was justified by achievement of similar if not better analytical performance over glassy carbon (GCE) and highly ordered pyrolytic graphite electrodes while others simply gave notice of poor kinetics characteristics^6^. Despite those contradicting observations, the optimal performance is attained with minimal pre-treatment of the active surface whereas for traditional solid electrodes this procedure can be relatively fastidious^7^. Furthermore, some modification of the active surface can always compensate for some lack of performance. For instance, nanostructuring with carbon-based materials increases the specific surface area, improves biocompatibility and allows functionalization for subsequent covalent attachment of bioentities^8^. When compared to more classical electrodes (glassy carbon, platinum, gold, etc.) PGEs make use of pencil mines available in stationery shops as polarizable material acquired at negligible cost in wide range of calibers.

The specificity for substrates turns the catalysis processes in enzyme biofuel cells less prone to chemical interferences. Hence, cells can operate without need of physical membrane separation between the anolyte and catholyte. Another attractive feature, particularly regarding alternative cleaner bio-batteries^9^, is that enzymes generally perform their catalysis function at rather mild conditions, as is the case of the physiologic chemical environment. A good example of this is the bilirubin oxidase (BOx) enzyme and the generation of overpotential in the cathodic oxygen reduction reaction (ORR). Its efficiency shows similarities or even outperforms the platinum catalysts^10^, but bypassing the drastic reaction conditions required by the last in the respective fuel cells^11^. The main issue is, in this context, how to assure direct electron transfer to the catalytic site of the BOx enzyme, whom is buried in an insulating glycoproteic shell^12^. The effective linking to the electrode active surface has been evidenced as major requirement in the attainment of bio-batteries and efficient enzyme biofuel cells^13–17^.

Herein, the positive features of PGEs bioelectrodes with direct electron transfer to the immobilized BOx are assed and critically compared regarding other prior approaches. As far as known, only two studies from the same group assessed the performance of PGEs as biocathodes in biofuel cells, but with immobilized laccase enzyme^10,11^. Prior to enzyme immobilization, the suitable PGE regarding the pencil hardness, pre-treatment of its surface and modification with carbon-based nanomaterials is selected after electrochemical evaluation. The immobilization procedure consisted of tethering BOx enzyme to multi-walled carbon nanotubes (MWCNTs) by pyrene-based succinimidyl ester compound (PBSE), as similarly performed by Ramasamy et al.^12^. The high electrocatalytic performance achieved with the proposed PGE indicates favorable and oriented bilirubin oxidase immobilization on the surface of nanotubes. It also allows foreseeing the possibility of being able of use as biocathode in miniaturized energy sources.

## Results

**Figure 1:**
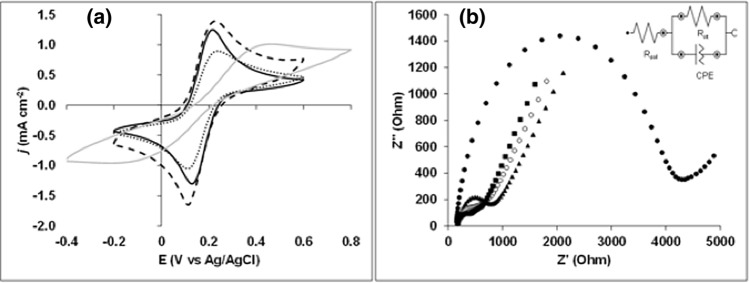
Characterization of PGE regarding the influence of pencil hardness and surface pre-treatment. (**a**) Cyclic voltammograms for a PGE type 4B (dashed line), HB (full black line), 4H (dotted line) and for a PGE HB without pre-treatment by polishing with alumina (full grey line). (**b**) Nyquist plots for PGE 4B (squares), HB (open circle), 4H (triangle) and for PGE HB without pre-treatment by polishing with alumina (full circle). Conditions for CV: scan rate of 50 mV s^−1^. Conditions for EIS: frequency 100,000 to 0.1 Hz, amplitude 0.01 V, potential set to OCP value. Electrolyte for both analysis: 5 mM Fe(CN)_6_^3−/4−^ with 0.1 M KCl, purged 15 min with N_2_.

PGEs with different clay/carbon ratios (hardness 4B, HB, and 4H) and extension of pre-treatment were stuck and levelled to the body of the reference electrode with rubber band for initial comparison by cyclic voltammetry CV and impedance spectroscopy EIS in Fe(CN)_6_^3−/4−^ electrolyte solution (Fig. 1). At first sight, the voltammetric signals obtained for 4B seemed better relative to HB (Fig. 1a). However, when taking into account the respective background currents, peaks were higher and more symmetrical for HB (i_pa_ = 1.38 mA cm^−2^; i_pc_ = − 1.44 mA cm^−2^) with an almost unitary i_pa_/i_pc_ ratio, followed by 4B (i_pa_ = 1.27 mA cm^−2^; i_pc_ = − 1.44 mA cm^−2^) and lastly by 4H (i_pa_ = 1.03 mA cm^−2^; i_pc_ = − 1.18 mA cm^−2^). Even noticing small differences in the response between mines of same hardness along repeated assays the relative behavior across the different types of PGE kept unchanged otherwise. PGE HBs had also better kinetics since peak-to-peak separation was lower (∆E_p_ = 0.09 V) in agreement with the results reported by Kariuki^18^. The assays also showed that without the polishing with alumina step, inferior kinetics (∆Ep = 0.64 V) and lower peak heights (i_pa_ = 0.63 mA cm^−2^; i_pc_ = − 0.57 mA cm^−2^) were obtained. For these PGEs, EIS performed at the open circuit potential of 0.145 V (vs Ag/AgCl) also confirmed a higher resistance to electron transfer (R_ct_ = 4000 Ω) translated by the semi-circle with a bigger diameter in the Nyquist plot (Fig. 1b).

The electrochemical response of the assayed electrodes using EIS spectroscopy can be mimicked by the general equivalent circuit depicted as inset of Fig. 1b. The numerical value corresponding to each element is further referred to in Table 1. As can be seen from the higher x to y axis amplitude in the Nyquist plot, semi-circles seemed depressed and centered below the abscissa axis in accordance to surface roughness and varying clay/carbon composition of PGEs. Thus, instead of representing through a capacitor the double-layer capacitance behavior, C_dl_, at the interface of PGE surface with solution, the constant phase element CPE is used. CPE values were calculated from Z_(ω)_ = q^−1^(*j* ω)^−n^, where q is a proportionality factor, *j* = − 1^1/2^, ω is the angular frequency and 0.8 < n < 1 describes the distorted capacitance behavior observed^19^. From the values stated in Table 1, it was also possible to conclude that alumina polishing allows general improvement of R_ct_ regardless the hardness of the pencil mines.

**Table 1:** Equivalent circuit component values for the different PGEs and surface pre-treatment.

PGE type	R_sol_ (Ω)	R_ct_ (Ω)	n	q (Ω^−1^ s^n^)
4H (pre-treated with alumina)	180	600	0.76	4.8 × 10^–6^
HB (pre-treated with alumina)	170	400	0.76	8.0 × 10^–6^
4B (pre-treated with alumina)	170	300	0.74	4.8 × 10^–6^
HB (without alumina pre-treatment)	100	4000	0.80	9.5 × 10^–7^

In fact, polishing procedures with alumina have been stated to improve the kinetics of the electrode in Fe(CN)_6_^3−/4−^^7^ by affecting the microstructure and roughness of the surface^20^, increasing the number of oxygen functionalities at its surface and reactiveness to certain ions^21^. Other authors have also confirmed the improvement in electron transfer upon electrochemical activation at fixed potentials of pristine PGE surface^22–25^. Assuming that careful polishing confers similar active surface smoothness regardless the mine hardness, the obtained values of *n* inferior to one (Table 1) corroborates the heterogeneous composition. In such circumstances, the decreasing R_ct_ values from 4H to 4B reflect progressive lower clay/carbon ratio. An increasing deviation regarding the Warburg impedance was observed in the same sequence due to non-uniform adsorption processes^19^. Based on these results the HB mines were selected for the remaining studies. They showed the best compromise between low R_ct_ values and almost unitary i_pa_/i_pc_ ratios in the voltammetric response to the Fe(CN)_6_^3−/4−^ probe.

**Figure 2:**
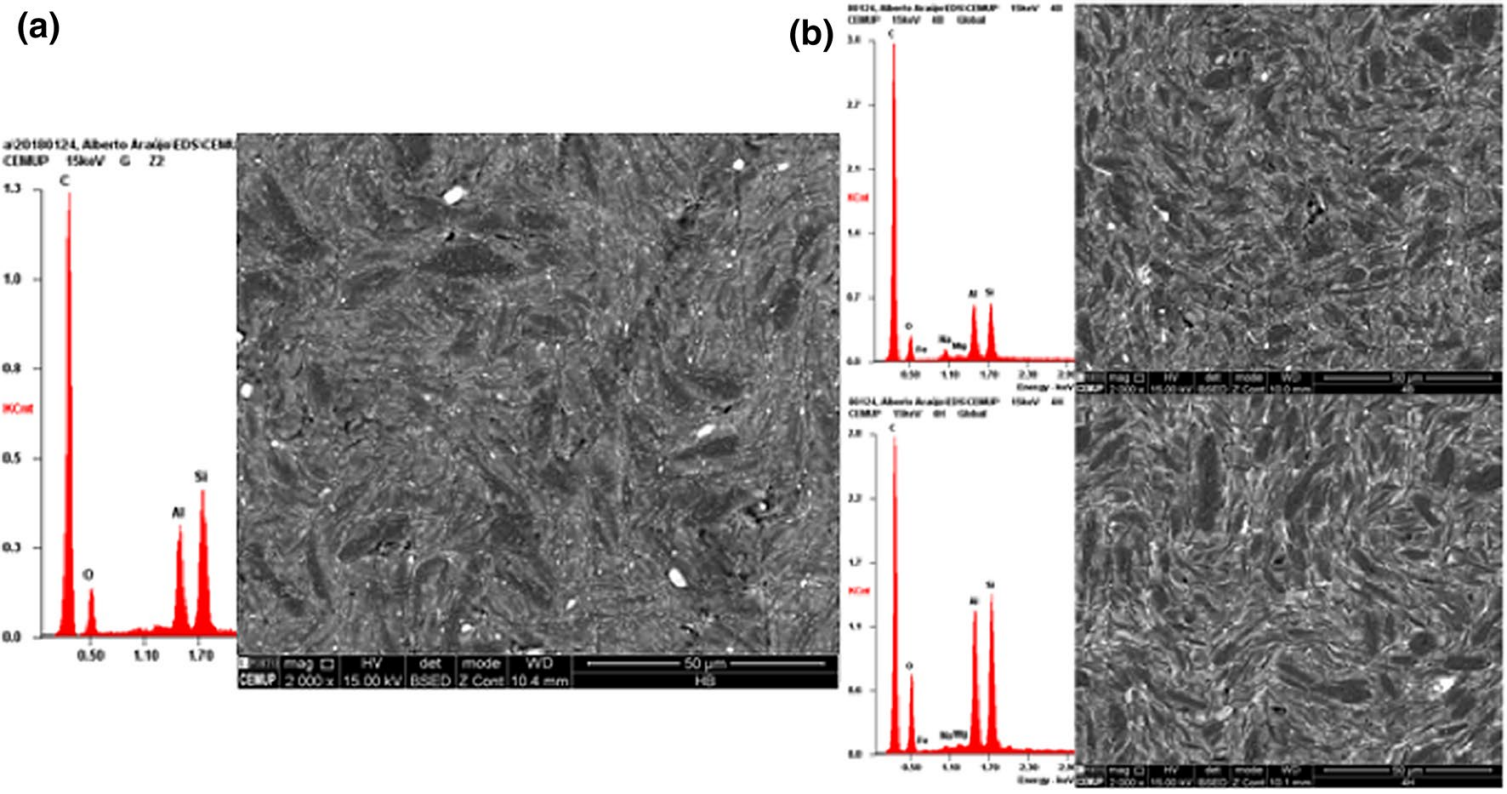
SEM–EDS micrographs of carbonaceous spot in (**a**) Staedtler HB mine and of (**b**) 4B and 4H transverse surfaces after mechanical polishing with 0.05 μm alumina aqueous slurry onto a polishing cloth followed by profuse washing with purified water.

The surface composition of the 3 types of pencil leads (4B, HB and 4H) as evidenced by energy-dispersive X-ray spectroscopy and scanning electron microscopy is highlighted in Fig. 2. The spectra reveal silicon, aluminum and oxygen as main components of clay. The respective micrographs depict an increasing number from 4H to 4B of dark gray carbon microcrystallites with approximately 2–10 µm length. The electroactive surfaces found congruently differ from the calculated geometric surface A_geom_ = 0.031 cm^2^ and were of A_eff_ = 0.037 ± 0.001 cm^2^ (n = 3) 4B, 0.034 ± 0.001 cm^2^ (n = 3) HB and 0.027 ± 0.002 (n = 3) cm^2^ 4H. The differences thus reflect both PGEs different clay/carbon ratios as well some microgranularity of the surface.

**Figure 3:**
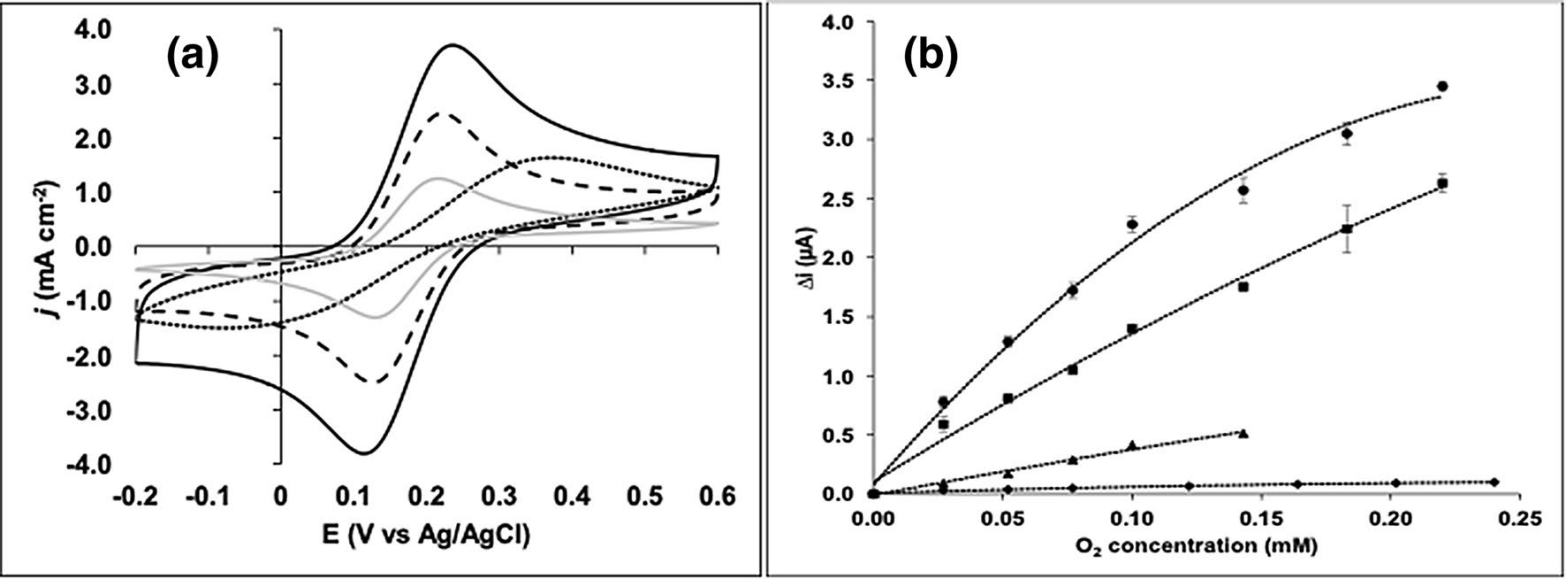
(**a**) Cyclic voltammograms of PGEs modified with carbon-based nanomaterials, namely PGE-rGO (full black line), PGE-MWCNT (dashed line), PGE-CB (dotted line) and bare PGE (full grey line). Conditions: scan rate 50 mV s^−1^, electrolyte: 5 mM Fe(CN)_6_^3−/4−^ with 0.1 M KCl, purged 15 min with N_2_; (**b**) Oxygen calibration curves for PGE-rGO-MWCNT-BOx (circles), PGE-MWCNT-BOx (squares), PGE-BOx (triangles) and PGE-rGO-BOx (diamonds): applied potential + 0.15 V; electrolyte solution: 10 mL of 0.1 M phosphate buffer pH 7.0, purged with N_2_ 15 min, 27.0, 52.0, 77.0, 100.0, 143.0, 183.0 and 220.0 μM O_2_.

Nanostructuration of electrodes with carbon-based materials provides enhanced electronic properties. Three different types of sp^2^ carbon commonly used for that porpoise were tested in PGEs with and without alumina pre-treatment and compared by CV again using the Fe(CN)_6_^3−/4−^ probe. Surprisingly, the physical stability of graphene or MWCNTs layers casted on surfaces previously polished with alumina was poor and often detached few instants after immersion in the probe solution. On contrary, the results obtained for PGE solely polished with sandpaper were significant. As depicted in Fig. 3a, surface modification with reduced graphene enabled voltammetric peaks three times higher (i_pa_ = 3.52 ± 0.14 mA cm^−2^; i_pc_ = -3.58 ± 0.14 mA cm^−2^, n = 3) relative to results obtained for HB previously polished with alumina. They also performed better regarding PGEs modified with MWCNT or carbon black despite the larger background current ascribed to the higher specific capacitance of graphene, 195 ± 6 F g^−1^^26^. The performance obtained after surface treatment with MWCNT (i_pa_ = 2.55 ± 0.09 mA cm^−2^; i_pc_ = − 2.68 ± 0.09 mA cm^−2^, n = 3) was also fairly good specially when compared to the bare and Vulcan modified PGEs (PGE-CB). As modifier, the carbon black was less efficient resulting in lower peaks (i_pa_ = 1.07 ± 0.08 mA cm^−2^; i_pc_ = − 1.08 ± 0.08 mA cm^−2^, n = 3) and higher ∆Ep (0.44 V).

### Characterization of BOx bioelectrode

In order to assess the importance of the modifications introduced by the use of reduced graphene and/or MWCNT, different biosensors were implemented, namely the PGE-BOx, PGE-MWCNT-BOx, PGE-rGO-BOx and PGE-rGO-MWCNT-BOx. The magnitude of respective amperometric responses to different molecular oxygen concentrations is plotted in Fig. 3b. A typical recording for the PGE-rGO-MWCNT-BOx at + 0.15 V showed prompt and large drifts caused by disruption of the quiescent condition with each O_2_ standard addition to the electrolyte solution. A short period of time after, the diffusion gradient is resumed and current signal steps to more negative values due to the biocatalytic reduction of the higher oxygen concentration. Unstable responses corresponding to about 5 µA were nevertheless observed at higher oxygen concentrations near the saturation threshold of the enzyme.

The additional data stating the performance observed for each bioelectrode is summarized in Table 2. As evidenced, the surface modification with the nanomaterials boosts the electroactive areas of PGEs with an effective area of 0.482 ± 0.17 cm^2^ for the PGE-MWCNT only surpassed by the 0.720 ± 0.25 cm^2^ for the PGE previously treated with rGO. The bioelectrode PGE-rGO-MWCNT-BOx presented the highest sensitivity to oxygen, with an average value for twelve assayed electrodes of 648 ± 51 µA mM^−1^ cm^−2^. Noteworthy, some units still exhibited 60% of that sensitivity value after being kept in the fridge immersed in buffer solution and sealed into falcon tubes for three months. The limit of detection (LOD), calculated as the 3.σ_blank_/slope, was of 1.7 µM and only matched by the LOD of the bioelectrode PGE-MWCNT-BOx. The interval of linear amperometric response to oxygen was nevertheless limited, 0.10 mM. The PBSE is a good tethering agent for the MWCNT support and fairly good even for the graphite crystallites in bare PGEs given the sensitivities obtained respectively for the PGE-MWCNT-BOx (332 ± 40 µA mM^−1^ cm^−2^, n = 6) and PGE-BOx electrodes (112 ± 14 µA mM^−1^ cm^−2^, n = 3), respectively. Thus, the more fastidious procedure for electrode modification with rGO is largely compensated by having a better surface for MWCNT-BOx immobilization since the sensitivity to O_2_ of the resulting bioelectrode almost doubles. The performance of the PGE implemented by direct linking BOx to rGO via PBSE was low (11 ± 3 µA mM^−1^ cm^−2^, n = 3). Both PGE-rGO-MWCNT-BOx and PGE-MWCNT-BOx had higher sensitivity to oxygen when compared with other biosensors referred in the literature^27–30^. Mousty et al. reported a biosensor with a sensitivity value of 470 µA mM^−1^ cm^−2^ under stirring conditions but with adding of the ABTS electron mediator^31^.

**Table 2:** Performance achieved for different configurations of the biocathodes. Electroactive surfaces determined before enzyme anchorage.

Biosensor configuration	Linear range (mM)	LOD (µM)	Sensitivity (µA/(mM.cm^2^))	Electroactive surface (cm^2^)
PGE-rGO-BOx	0.27	71.0	11	0.572
PGE-BOx	0.15	4.0	112	0.034
PGE-MWCNT-BOx	0.31	1.6	332	0.482
PGE-rGO-MWCNT-BOx	0.10	1.7	648	0.720

Electroactive surfaces determined before enzyme anchorage.

**Figure 4:**
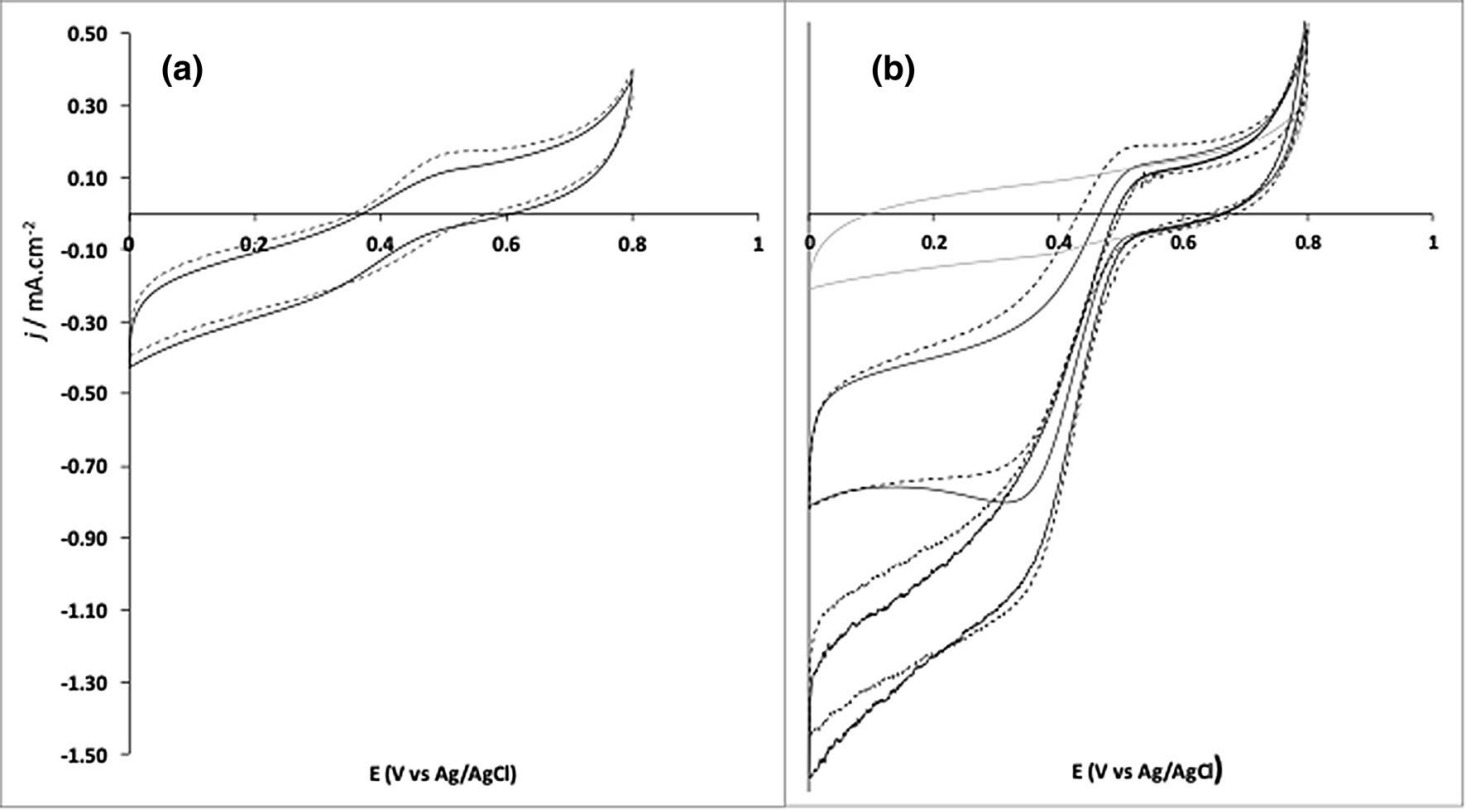
Response of the electrodes to the presence of O_2_. (**a**) BOx immobilized by adsorption on the surface PGE-rGO-MWCNT (full line) and further adding of 0.5 mM ABTS to the electrolyte solution (dashed line). (**b**) BOx immobilized by crosslinking to the surface of PGE-rGO-MWCNT (full line) and further adding of 0.5 mM ABTS to the electrolyte solution (dashed line); both currents increase with solution magnetic stirring at 600 r.p.m. Full grey line correspond to the response of PGE-rGO-MWCNT-Box in N_2_ saturated electrolyte. Other conditions: scan rate 10 mV s^−1^. Electrolyte: 10 mL of 0.1 M phosphate buffer pH 7.0; electrode surface area: 0.034 cm^2^.

**Figure 5:**
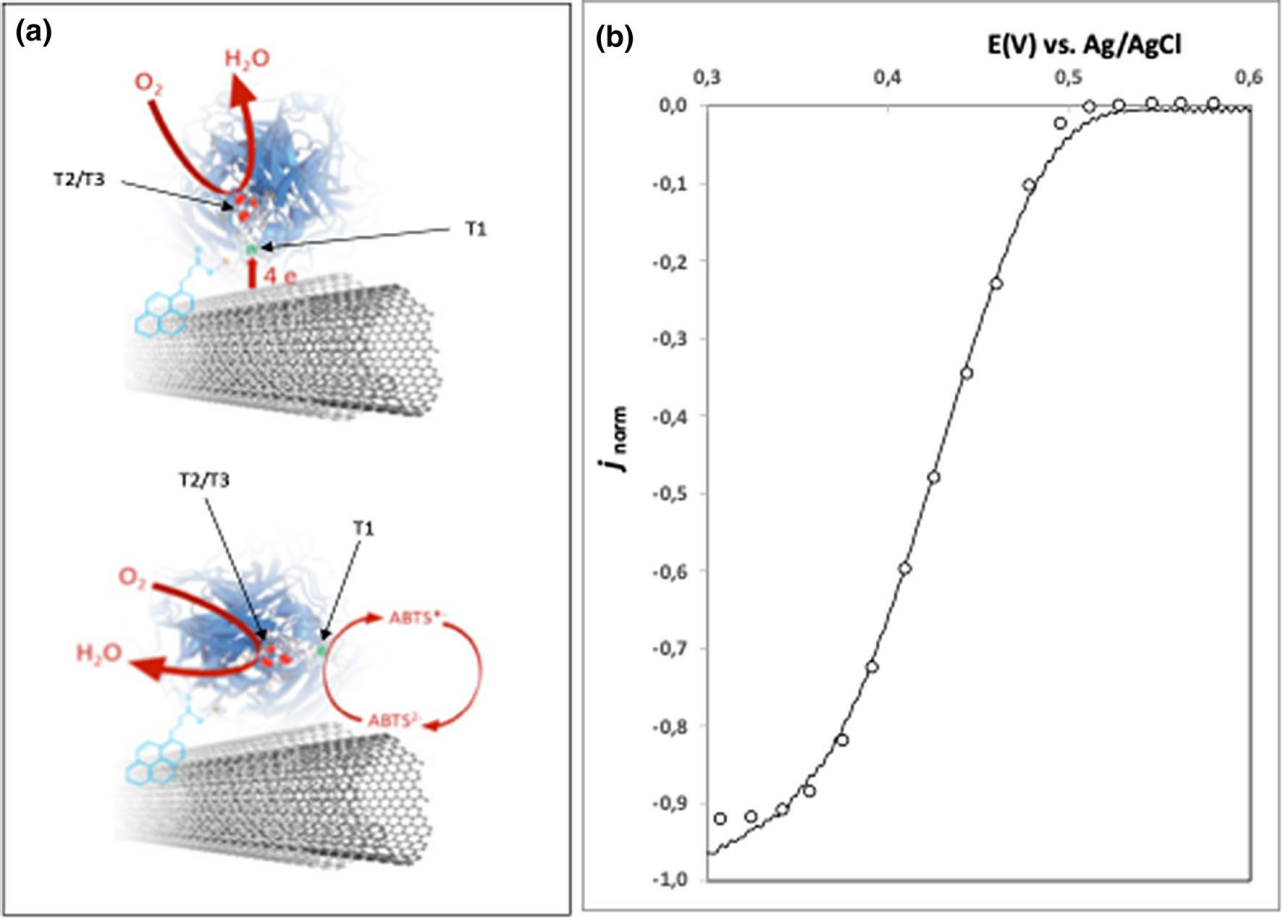
(**a**) Scheme of two possible orientations for the anchored BOx on MWCNT. On the top, direct electron transfer is enabled to the T1 catalytic site. On the bottom, the electron transfer is mediated by ABTS. (**b**) Non-linear least squares regression of Armstrong’s basic model on PGE-rGO-MWCNT-BOx normalized voltammogram of Fig. 4b under stirring conditions: results simulated by the fitted model are represented by open circles.

Further CV analysis was performed to assess the best scheme for immobilization of the BOx, i.e. adsorbed or tethered through PBSE. Figure 4 shows the catalytic response of the biosensors PGE-rGO-MWCNT-Box (adsorbed) and PGE-rGO-MWCNT-Box (tethered) in quiescent solution and with stirring at 600 r.p.m, with and without adding the ABTS mediator. The reaction catalyzed by BOx leads to O_2_ reduction after internal electronic flow between the T1 catalytic site and the complex T2/T3 redox center. If BOx is efficiently oriented on the MWCNT surface (Fig. 5a top), the electrode acts itself as artificial substrate and donates electrons to the T1 catalytic site of the enzyme. In both schemes tested, the displacement of the voltammetric curves towards more negative values of current evidences this direct electron transfer mechanism (DET). In quiescent solution, the reduction onset starts at about 0.45 V for the electrode with tethered BOx and reaches a maximum current density of − 0.75 mA cm^−2^ at 0 V. Both reverse and forward scans also share similar shape with other BOx based biosensors that employed PBSE as a tethering agent^12,32–34^. However, the maximum current achieved at 0 V was 2.3 times higher when compared to a similar biosensor based on Toray carbon paper as transducer (0.325 mA cm^−2^)^12^. The current values were also higher relative to PGE-rGO-MWCNT-Box (adsorbed), and less dependent of applied potential below 0.34 V.

De-an Li and collaborators adsorbed BOx from *Myrothecium verrucaria* on glassy carbon and on edge-plane pyrolytic graphite (EPPG) electrodes^35^. When studying the DET process, they ascribed different enzyme orientations on the surface of electrodes to explain four different redox potential peaks corresponding to the catalytic sites T1 and T2/T3. After activation of BOx from its oxidized resting form with the applied lower potentials of the first CV scan, the higher redox potential of the catalytic T1 site (E^0^_T1_ = 0.70 V vs NHE) relative to the complex T2/T3 redox center (E^0^_T2/T3_ = 0.49 V vs NHE) became evident in the course of the second scan^36–38^. Noteworthy, the voltammograms obtained in the present work only revealed two small reversible peaks at 0.50 V and 0.38 V (E_1/2_ = 0.46 V vs Ag/AgCl, 0.1 M KCl) in the absence of oxygen. The absence of peaks at lower potentials, corresponding to the T2/T3 redox center, was indicative of preferential enzyme orientation over the modified electrode surface. This aspect is better evidenced when an excess of ABTS mediator is added to the electrolyte solution, here up to the concentration of 0.5 mM (Fig. 4, dashed lines). Inefficient direct electron transfer to the T1 site of the enzyme would be compensated by additional supply of electrons from ABTS (Fig. 5a bottom). However, this was not the case since the voltammograms obtained without and after ABTS addition show almost similar cathodic waves either in quiescent conditions and when the oxygen concentration gradient was disrupted by solution stirring. On other hand, the voltammograms were not similar for the PGE-rGO-MWCNT-Box (adsorbed) where the electron shuttling by ABTS becomes apparent after stirring or by the enzyme detachment.

Other approaches^39–43^ were described regarding oriented immobilization of BOx on modified surfaces of electrodes and for some of them^39,40,43^ the DET quality was evaluated by two terms, *βd*_*0*_ and *p*, according a mathematical model proposed by the group of Armstrong et al.^43^:

$$j = \frac{{j_{\lim } }}{{\beta d_{0} }}\frac{{e_{1} - e_{2} }}{{1 + e_{1} }}\ln \frac{{pe_{1}^{{\alpha_{c} }} + (1 + e_{1} )}}{{pe_{1}^{{\alpha_{c} }} + (1 + e_{1} )\exp ( - \beta d_{0} )}}$$

with *j* and *j*_*lim*_ representing respectively the normalized density current and limiting current, α_c_ the transfer coefficient (the 0.5 value being assumed throughout), *e*_1_ and *e*_2_ the driving force for interfacial electron transfer and introduced biocatalytic bias. The *βd*_*0*_ term mirrors different orientations the enzyme molecules may adopt while maintaining electron exchange with the electrode. The second term *p*, is the ratio between the enzymatic catalytic rate and the electron transfer rate from catalytic site to the electrode. The author of the model reported the respective values of about *βd*_*0*_ = 26.2 and *p* = 51.5 for a pyrolytic graphite edge electrode modified with naphthyl-2-carboxylate functionalities for enhanced adsorption of BOx from *Myrothecium verrucaria*. For similar pyrolytic graphite electrodes alternatively modified with pristine MWCNT, carboxyl- or amine functionalized MWCNT^39^, the *βd*_*0*_ term revealed pH dependence. Much narrow distribution of orientations (4.8 < *βd*_*0*_ < 7.6; *p* = 0.16) was observed for the adsorption on negatively-charged MWCNT-COOH in the pH range from 5 to 7.8^39^. Values of *βd*_*0*_ higher than 20 (and *p* = 1.63) were observed for MWCNT-NH_2_ pH from 3 to 7, congruent with electrostatic repulsion between the protonated amine group and the positive charge of copper at T1. Recently, the surface of a gold electrode was made porous (i.e. with spherical pores) after anodization with glucose electrolytic solution, being the BOD enzyme afterwards adsorbed^40^. Very low values were obtained (*βd*_*0*_ = 2; *p* = 0.5) but authors considered n = 1 as rate determining step number for the interfacial electron transfer for the T1 site, while the overall ORR process n = 4 was considered in previous works. The forward scan of plot obtained under stirring in Fig. 4b was subtracted from its blank counterpart (cathodic wave obtained after immersion in 10 mL of 0.1 M phosphate buffer pH 7.0, purged with N_2_ for 15 min). After results normalization the non-linear least squares regression provided the values of *βd*_*0*_ = 9.64 and *p* = 0.65 with a predicted E^0^_T1_ = 0.48 V vs Ag/AgCl, Fig. 5b. The obtained *e*_2_ > 3.67 × 10^9^ also evidenced irreversible reduction of O_2_. The term regarding dispersion of orientations is a bit higher than reported for MWCNT-COOH but was not obtained with a rotating disk electrode. The *p* value below one enables to conclude that the BOx catalytic rate is the determining step and not the DET process.

## Discussion

**Figure 6:**
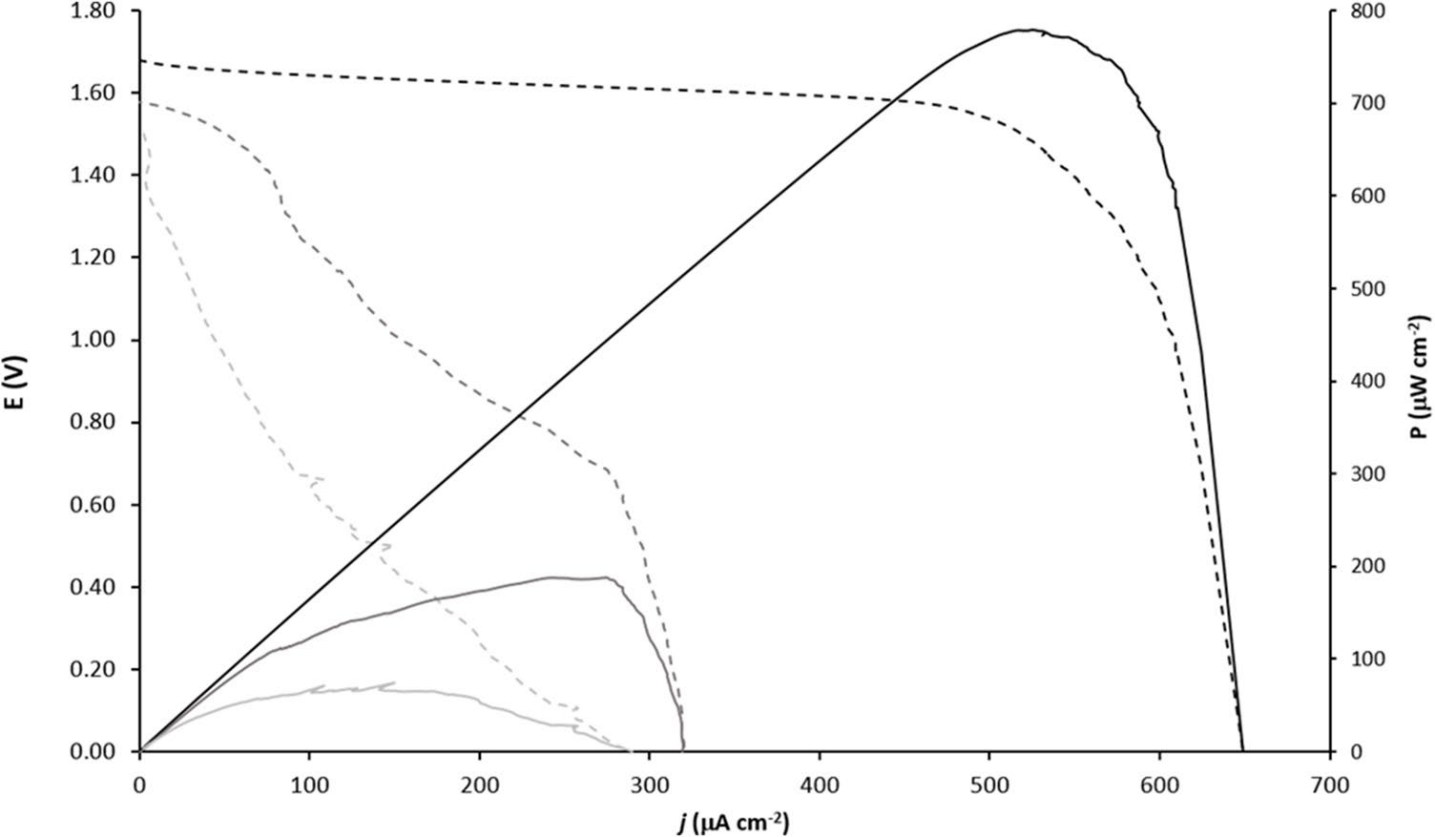
Polarization (dashed line) and power (full line) curves obtained with PGE-rGO-MWCNT-BOx electrode as biocathode and a zinc electrode as anode in the absence of O_2_ (light grey), air saturated (dark grey) and O_2_ saturated electrolyte. Conditions: 10 mL of 0.1 M phosphate buffer pH 7.0; electrode surface area before nanostructuring: 0.034 cm^2^.

The establishment of direct electron transfer between PGE and BOx makes possible the application of the bioelectrode as biocathode in membraneless miniaturized biofuel cells and bio-batteries. Therefore, the bioelectrode with the highest sensitivity for oxygen (PGE-rGO-MWCNT-BOx) was selected and connected with a zinc rod anode for the bio-battery experiments. Figure 6 depicts the polarization curves after linear scanning voltammetric (LSV) measurements at 1 mV s^−1^. In the oxygen saturated electrolyte, the open circuit potential (OCP) of the biofuel cell corresponded to 1.68 V and the maximum current density to 650 µA cm^−2^. These results enable the calculation of a maximum power density of 775 ± 41 µW cm^−2^ (n = 5) corresponding to an increase of about 400% when compared to the performance in air saturated electrolyte.

As far as known, the only PGE-based biocathode found in the literature was developed by Kashyap et al.^10^. This biocathode resorted to the mediator ABTS to facilitate the electron transfer between laccase and the polyaniline-MWCNT modified PGE. The achieved OCP was of 0.58 V (vs. Ag/AgCl) and the maximum current density was of 296 µA cm^−2^. Other BOx biocathodes using PBSE as a tethering agent have also been studied however using different transducers^12,32,33^. In the approach from Strack et al.^32^, the MWCNT buckypaper biocathode generated an OCP of 0.48 V and produced about 200 µA cm^−2^ of maximum current density. In turn, Lopez et al.^33^ immobilized the enzyme substrate, bilirubin or its artificial analogs, in an MWCNT/Nafion modified GCE to modulate BOx orientation. For either bilirubin or the analogs, the OCP was around 0.5 V and maximum current density corresponded to about 300 µA cm^−2^ in the first case and about 750 µA cm^−2^ for the analog 2,5-dimethyl-1-phenyl-1H-pyrrole-3-carbaldehyde.

## Conclusions

In the present work, we have prepared a simple and viable O_2_ biocathode. The availability and easy fabrication of the PGE conjugated with the simplicity of the enzyme immobilization procedure allows its use in membraneless miniaturized cleaner power sources. Commonly used pre-treatment schemes, such as careful alumina polishing and smoothing, enhanced general electrochemical response of PGEs but also jeopardize the physical robustness of surface modifying by carbon-based nanomaterials. Previous PGE modification with reduced graphene increases the preparation time of PGE bioelectrodes but also increases the sensitivity towards oxygen by being a better support for MWCNT immobilization. Tethering of BOx on MWCNT using PBSE provides a long lasting bioelectrode with efficient DET feature and with physical stability to be used in stirred fuel solutions.

## Methods

The enzyme bilirubin oxidase from *Myrothecium verrucaria* (8 U/mg of solid) was acquired from Sigma-Aldrich being the activity tested before use according the procedure provided by the seller. Aliquot solutions of bilirubin oxidase (8 U/mL) were made by dissolving the solid in 0.01 M phosphate buffer solution pH 7.0 and stored at – 20 °C until use. The 2,2′-Azino-bis(3-ethylbenzothiazoline-6-sulfonic acid) diammonium salt (ABTS), graphene oxide (4 mg/mL dispersion in water), hydrochloric acid 37%, MWCNT (carboxylic acid functionalized), potassium hexacyanoferrate (II) trihydrate, potassium hexacyanoferrate (III), potassium phosphate dibasic, potassium phosphate monobasic were also from Sigma-Aldrich. Dimethylformamide (DMF) was acquired from ROMIL Chemicals (Cambridge, UK).

All experiments were performed in a typical three-electrode electrochemical cell composed of an Ag/AgCl (KCl, 0.1 M) reference electrode (Metrohm, Ref. 6.0727.000), a platinum rod as counter electrode and the PGE sensor/biosensor as working electrode. The voltammetric and amperometric experiments were accomplished with a potentiostat Metrohm, model Autolab PGSTAT10, controlled by GPES v3.9 software (Herisau, Switzerland). Electrochemical impedance spectroscopy (EIS) experiments were performed with an Autolab PGSTAT204, model FRA32M, controlled by NOVA v1.10.1.9 software. An equimolar 5 mM potassium hexacyanoferrate, Fe(CN)_6_^3−/4−^ in 0.1 M of KCl solution (resistance of the solution, R_sol_ = 180 Ω) was used in the evaluation of PGEs without immobilized enzyme. A 0.1 M potassium phosphate buffer solution, pH 7.0, was used as electrolyte solution, alternatively purged with N_2_ or oxygenated for 15 min, along experiments with the bioelectrode. For the amperometric measurements of O_2_, known volumes of oxygen saturated solution (1.1 mM O_2_, partial pressure of 0.79594 atm at 293.15 K) were added to the electrochemical cell containing 10 mL of N_2_ purged electrolyte solution. The oxygen concentrations were additionally checked with the Sension + DO6 field kit from Hach (Loveland, Colorado). All potentials presented throughout the text are referenced to Ag/AgCl.

In order to assess the influence of pencil hardness and pre-treatment on the electrochemical performance, each mine 4H, HB or 4B with 2 mm diameter (Staedtler) was put in contact with the inner copper wire of a shielded coaxial cable and the electric contact was isolated with flexible Tygon polymer sleeve. A transversal cut near the distal ending enabled the access to a pristine PGE surface. This surface was polished mechanically using sandpaper (P1200) and profusely washed with distilled water afterwards. The exposed surface of some pencil mines was additionally smoothed with alumina 1.0 µm and 0.05 µm in a polishing cloth, rinsed with water and sonicated 2 min in ethanol. The SEM–EDS exam was performed using a High resolution (Schottky) Environmental Scanning Electron Microscope with X-Ray Microanalysis and Electron Backscattered Diffraction analysis: Quanta 400 FEG ESEM/EDAX Genesis X4M.

Only the pencil mines HB polished with sandpaper were used for biocathode implementation. The surface was modified with 10 μL of graphene oxide (1 mg mL^−1^) then electrochemically reduced along 50 scans performed within − 1.2 V and 0.8 V at 50 mV s^−1^ in 0.1 M Na_2_SO_4_ solution. These electrodes are henceforth referred as PGE-rGO. About 6 μL of 1 mg mL^−1^ MWCNT in DMF were dripped over each PGE-rGO surface and left to dry at 50 °C for one hour. Conditioning with 10 mM PBSE in DMF solution for 1 h and further washing for a few seconds in 0.01 M phosphate buffer solution (pH 7.0) was proceeded. In this way, the irreversible π–π stacking of the pyrenyl moiety of each PBSE molecule on the aromatic-like walls of MWCNT was formed. Finally, the electrodes were immersed in 0.5 mg mL^−1^ enzyme BOx solution for another hour and similarly rinsed with the phosphate buffer. The succinimidyl leaving group of PBSE allowed the formation of a covalent amide bond after reaction between the terminal carboxyl with an amine group of the enzyme polypeptide chain^12^. The bioelectrodes thus prepared are henceforth designated as PGE-rGO-MWCNT-BOx.

In order to calculate the current densities referred along the text, the active surface area of mechanically polished PGEs was determined from the peak current vs. scan rate plot obtained by cyclic voltammetry and applying the Randles–Sevcik equation for reversible a process as described in Ref.^44^. The prepared concentration of the hexacyanoferrate system of 5 mM was assumed as bulk concentration and the diffusion coefficient of hexacyanoferrate ion was set to 0.65 × 10^–5^ cm^2^ s^−1^.

For comparison purposes, other nanostructured PGEs were prepared. Instead of rGO, some were modified with 10 μL MWCNT (1 mg mL^−1^ in DMF), others with 10 μL carbon black Vulcan XC72 (1 mg mL^−1^ in water) and designated as PGE-MWCNT and PGE-CB, respectively. The step regarding the modification with the tethering agent was also omitted in the bioelectrodes later on referred as PGE-rGO-MWCNT-Box (adsorbed).
